# Magnitude and contributing factors responsible for premature greying of hair: A cross-sectional study

**DOI:** 10.6026/973206300211658

**Published:** 2025-06-30

**Authors:** Ransingh Tanwar, Abhay Kumar, Vikas Rangare, Chandra Shekhar Jaiswal, Dileep Dandotiya

**Affiliations:** 1 Department of Dermatology, Venerology and Leprosy, GMC Mandsaur, Madhya Pradesh, India; 2 Department of ENT, CIMS Chhindwara, Madhya Pradesh, India; 3 Department of Medicine, CIMS Chhindwara, Madhya Pradesh, India; 4 Department of Dermatology, Venereology and Leprosy, Chhindwara Institute of Medical Sciences, Chhindwara, Madhya Pradesh, India; 5 Department of Community Medicine, CIMS Chhindwara, Madhya Pradesh, India

**Keywords:** premature canities, prevalence, risk factors, vitamin B12, smoking, oxidative stress, India

## Abstract

The magnitude of PGH and identify modifiable and non-modifiable contributing factors in an academic medical setting is of interest. We recruited 520 consecutive dermatology out-patients. PGH was defined as the presence of ≥5 grey hairs before age 25 in Asians. Nearly one in four young adults attending our teaching hospital had PGH. Genetic predisposition, modifiable lifestyle factors and correctable micronutrient deficiencies independently influenced risk. Thus, we report early behavioural and nutritional interventions integrated into student-health clinics could attenuate the psychosocial burden of PGH.

## Background:

Premature greying of hair (PGH) or premature canities is being reported with increasing frequency in young adults worldwide, but robust epidemiological data from South-Asian teaching hospitals remain limited [1, 2]. Human scalp hair normally begins to grey after the fourth decade when melanocyte activity in hair follicles declines gradually with age [3]. Premature greying of hair (PGH), alternatively termed premature canities, denotes depigmentation occurring a decade or more earlier than the race-specific average-*i.e.* before 20 years in Caucasians, 25 years in Asians and 30 years in Africans [2]. Though traditionally considered a benign cosmetic concern, PGH is increasingly recognised as a cutaneous signpost of systemic dysmetabolism, oxidative stress and accelerated biological ageing [4]. Reported prevalence varies widely from 2 % in adolescents of mainland China to 32 % among medical students in North India [1, 5]. Heterogeneity arises from divergent definitions, population genetics, environmental exposures and study designs. A 2024 Saudi survey of 1 831 undergraduates quantified PGH in 36 % and identified low ferritin, vitamin D insufficiency, family history and mental stress as correlates [2]. Similarly, Saoji *et al.* observed 27 % prevalence among 1 062 Indian under-25s and confirmed smoking and sedentary lifestyle as independent risks [1]. Beyond lifestyle, mounting molecular evidence implicates reactive oxygen species that overwhelm intrinsic antioxidant systems, leading to melanocyte apoptosis and depletion of stem-cell reservoirs [6]. Nasrin *et al.* demonstrated that disruption of NOTCH signalling, pivotal for melanocyte maintenance, precipitated early greying in murine models, underscoring a genetic-epigenetic interface [3]. Cardio-metabolic sequelae associated with ectopic greying further augment clinical relevance. Large case-control analyses have linked premature canities and androgenetic alopecia with sub-clinical atherosclerosis and coronary artery disease in men less than 40 years [7]. A cross-sectional Korean study involving 1 929 healthy adults reported an adjusted odds ratio of 1.43 for metabolic syndrome in PGH versus age-matched controls [8]. Proposed mechanisms include chronic low-grade inflammation, altered lipid peroxidation, endocrine dysregulation and micro-nutrient deficits (vitamin B12, folate, biotin and iron, copper) that co-drive melanocyte exhaustion [4]. Therefore, it is of interest to describe the magnitude and contributing factors responsible for premature greying of hair.

## Materials and Methods:

This descriptive analytical study employed a cross-sectional design at the dermatology OPD of M.G. Medical College & M.Y. Hospital, Indore (M.P.), India (tertiary-care teaching institute). Consecutive patients aged 18-40 years presenting between 1 January and 31 December 2024 were screened. Exclusion criteria were: (a) syndromic disorders known to cause canities (*e.g.* vitiligo, Werner's syndrome), (b) ongoing chemotherapy, (c) recent (<3 months) hair-colour treatment, (d) pregnancy or lactation, and (e) unwillingness to consent. Sample size (n = 520) was calculated using single-proportion formula with anticipated prevalence 27 % , 95 % confidence, 5 % absolute precision, and 10 % non-response inflation. A structured questionnaire captured age, sex, occupation, physical activity (IPAQ-short), smoking and alcohol history, sleep duration, psychosocial stress (Perceived Stress Scale-10), and dietary patterns. Family history of PGH in first-degree relatives was recorded. Anthropometry followed WHO STEPS. Venous blood assays included serum vitamin B12, ferritin, TSH, fasting lipids and glucose. PGH was diagnosed by two trained dermatologists when ≥5 depigmented hairs were observed on a 1 cm^2^ scalp area using dermatoscopy in individuals ≤25 years, or any visible grey hair in 26-40-year group fulfilling race-adjusted definition. Data were entered in EpiCollect-5 and analysed using SPSS v29. Continuous variables (mean ± SD or median [IQR]) were compared with Student's t or Mann-Whitney U as appropriate; categorical variables with χ^2^ test. Variables with p < 0.20 in bivariate analysis entered a multivariable binary logistic regression (backward stepwise). Model diagnostics included Hosmer-Lemeshow goodness-of-fit and variance inflation factors. Two-tailed p < 0.05 denoted statistical significance. Institutional Ethics Committee approved the protocol (IEC/2023/DERM/117). Written informed consent was obtained from all participants.

## Results:

A total of 548 eligible subjects were approached; 520 consented (response rate 94.9 %). Mean age was 27.4 ± 5.8 years; 51.2 % were male. Table 1 (see PDF) summarises baseline characteristics. Prevalence of PGH was 23.8 % (n = 124). On unadjusted analysis (Table 2 - see PDF), PGH was significantly associated with male sex (χ^2^ = 6.2, p = 0.012), ever-smoking (χ^2^ = 18.9, p < 0.001), high perceived stress (χ^2^ = 10.7, p = 0.001), vitamin B12 deficiency (χ^2^ = 25.4, p < 0.001), low ferritin, and positive family history (χ^2^ = 56.3, p < 0.001). Multivariable logistic regression (Table 3 - see PDF) retained five independent predictors: family history (aOR 3.5), smoking ≥10 pack-years (aOR 2.1), vitamin B12 deficiency (aOR 2.8), high stress (aOR 1.9) and sub-clinical hypothyroidism (aOR 1.6, borderline significance). Variance inflation factors were < 1.9, indicating negligible collinearity. Hosmer-Lemeshow p = 0.42 supported good model fit. Model including interaction terms between smoking and vitamin B12 deficiency improved likelihood ratio by 4.3 (p = 0.038), suggesting synergism. Sensitivity analyses restricted to 18-25-year subset yielded similar effect sizes.

## Discussion:

Our study revealed that almost one quarter (23.8 %) of young adults attending a tertiary dermatology clinic exhibited PGH, corroborating recent Indian and Middle-Eastern surveys that place prevalence between 24 % and 36 % [1, 2]. The upward trend with ageing within the 18-40-year window mirrors the age-stratified gradient documented by Almudimeegh *et al.* reinforcing that PGH is a progressive, not static, phenomenon [2]. Consistent with the polygenic inheritance hypothesis, a positive family history conferred the strongest risk (aOR 3.5). Earlier genome-wide association studies have linked variants near the IRF4, BCL2 and KITLG loci with early greying [3, 6] underscoring the role of melanocyte stem-cell exhaustion pathways. However, genetic predisposition alone cannot explain the rapid secular increase described among Gen-Z cohorts on social media reports [9] highlighting the contribution of environment-gene interactions. Smoking emerged as the most important modifiable lifestyle factor (aOR 2.1). Cigarette smoke accelerates follicular oxidative stress, elevates hydrogen peroxide and down-regulates anti-oxidative enzymes such as catalase [4, 10]. Our data align with a pooled estimate from a 2021 meta-analysis where smokers had a 2.05-fold increased odds of PGH [4]. The synergistic interaction observed between smoking and vitamin-B12 deficiency further amplifies risk [5]. High perceived stress doubled the odds of PGH. Chronic psychological stress augments sympathetic outflow and norepinephrine release in the niche, provoking depletion of pigment-producing stem cells as elegantly demonstrated in murine models by Zhang *et al.* [11]. Clinically, pandemics of academic and career anxieties might partly explain the heightened PGH burden in university settings [1]. Stress management interventions may therefore hold promise [12]. Micronutrient insufficiency was notable only for vitamin-B12, not ferritin, after adjustment. Although iron plays a role in tyrosinase activity, conflicting evidence and gender-biased dietary patterns warrant larger nutritional studies. Similarly, sub-clinical hypothyroidism showed a borderline relationship, reflecting the modulatory effect of thyroid hormones on melanocyte proliferation. Endocrine screening in PGH patients, particularly females, appears judicious given overlaps with autoimmune thyroiditis [13]. Our findings have clinical and public-health implications. First, dermatologists should adopt a holistic approach, screening for smoking, stress, nutritional deficits and thyroid dysfunction in young patients troubled by grey hair. Second, targeted health-promotion campaigns in colleges emphasising smoking cessation and balanced diets rich in B-complex vitamins may delay onset. Third, the genetic preponderance underscores the need for family counselling and prospective studies evaluating gene-environment interplay. Comparison with previous work reveals both concordances and discrepancies. Saoji*et al.* reported physical inactivity as an independent predictor, which lost significance in our fully adjusted model-potentially due to differential measurement of activity (IPAQ objective categories vs self-perception) [1]. Contrary to Korean data linking metabolic syndrome to PGH [8], our cohort showed no association after controlling for BMI, possibly reflecting ethnic or dietary differences. Strengths of this study include rigorous scalp examination by dermatologists, objective biochemical assays, adjustment for multiple confounders, and adherence to STROBE guidelines. Limitations must be acknowledged: (i) single-centre design may limit generalisability; (ii) cross-sectional nature precludes causal inference; (iii) recall bias in self-reported stress and smoking; (iv) absence of oxidative stress biomarkers (*e.g.*, malondialdehyde) precluded pathophysiological correlation [10]. Future research should adopt longitudinal designs to ascertain temporal relationships and explore epigenetic modifications associated with lifestyle changes. Randomised trials evaluating vitamin-B12 repletion, antioxidant supplementation, and structured stress-reduction could clarify reversibility of PGH documented sporadically in case reports [12]. Integrating trichoscopic metrics, hair mineral analysis and genomic sequencing will yield a multidimensional understanding of premature hair ageing.

## Conclusion:

Premature greying affected nearly one quarter of young adults at our teaching institute. Genetic susceptibility remains paramount; however, modifiable exposures-tobacco use, psychological stress and vitamin-B12 deficiency-substantially increased risk. Incorporation of lifestyle counselling, mental-health screening and micronutrient optimisation into dermatology and student-health clinics could mitigate both cosmetic concern and potential systemic sequelae. Multicentre prospective studies are warranted to validate these determinants and inform evidence-based preventive strategies.
